# Neural Mechanism of Altered Limb Perceptions Caused by Temporal Sensorimotor Incongruence

**DOI:** 10.3389/fnbeh.2018.00282

**Published:** 2018-11-20

**Authors:** Osamu Katayama, Tatsuya Tsukamoto, Michihiro Osumi, Takayuki Kodama, Shu Morioka

**Affiliations:** ^1^Department of Neurorehabilitation, Graduate School of Health Sciences, Kio University, Nara, Japan; ^2^Department of Rehabilitation, Watanabe Hospital, Aichi, Japan; ^3^Department of Undergraduate School of Health Sciences, Kio University, Nara, Japan; ^4^Department of Neurorehabilitation Research Center, Kio University, Nara, Japan; ^5^Department of Physical Therapy, Graduate School of Health Sciences, Kyoto Tachibana University, Kyoto, Japan

**Keywords:** temporal sensorimotor incongruence, peculiarity, altered limb perceptions, electroencephalogram, exact low-resolution brain electromagnetic tomography (eLORETA), eLORETA-ICA, supplementary motor area, parietal association area

## Abstract

Previous studies have demonstrated that patients with strokes or pathological pain suffer distorted limb ownership and an inability to perceive their affected limbs as a part of their bodies. These disturbances are apparent in experiments showing time delays between motor commands and visual feedback. The experimental paradigm manipulating temporal delay is considered possible to clarify, in detail, the degree of altered limb perception, peculiarity and movement disorders that are caused by temporal sensorimotor incongruence. However, the neural mechanisms of these body perceptions, peculiarity and motor control remain unknown. In this experiment, we used exact low-resolution brain electromagnetic tomography (eLORETA) with independent component analysis (ICA) to clarify the neural mechanisms of altered limb perceptions caused by temporal sensorimotor incongruence. Seventeen healthy participants were recruited, and temporal sensorimotor incongruence was systematically evoked using a visual feedback delay system. Participants periodically extended their right wrists while viewing video images of their hands that were delayed by 0, 150, 250, 350 and 600 ms. To investigate neural mechanisms, altered limb perceptions were then rated using the 7-point Likert scale and brain activities were concomitantly examined with electroencephalographic (EEG) analyses using eLORETA-ICA. These experiments revealed that peculiarities are caused prior to perceptions of limb loss and heaviness. Moreover, we show that supplementary motor and parietal association areas are involved in changes of peculiarity, limb loss, heaviness and movement accuracy due to temporal sensorimotor incongruence. We suggest that abnormalities in these areas contribute to neural mechanisms that modify altered limb perceptions and movement accuracy.

## Introduction

To initiate an action, the motor command necessary to achieve the desired outcome is generated first. A copy of this motor command has been described as an efference copy (Von Holst and Mittelstaedt, 1950/1973) or corollary discharge (Sperry, 1950) and is compared with the sensory consequences of the ensuing movements (Desmurget and Grafton, 2000; Wolpert and Ghahramani, 2000). This process is controlled by the comparator model, in which a comparison between desired and estimated states allows the calculation of motor error and immediate motor correction (Synofzik et al., 2008).

Sensorimotor incongruence can lead to pathological pain such as phantom limb pain (Harris, 1999). Ramachandran and Rogers-Ramachandran (1996) suggested that phantom limb pain after amputation is caused by incongruence between motor intention and sensory feedback. The loss of limb ownership and motor disturbance are important clinical features in pathological pain patients (Schwoebel et al., 2002; Moseley, 2005; van Hilten et al., 2005). Recent studies have also shown that experimental sensorimotor incongruence causes pain and other symptoms in people with pathological pain (McCabe et al., 2007; Daenen et al., 2012). Furthermore, previous study has suggested that patients with apraxia lack a general concept of the body which is necessary to mediate the translation of an action seen on the model into the position on the patients (Goldenberg, 1995). It was also demonstrated that loss of limb ownership is frequently observed in stroke patients (Feinberg et al., 2010; Zeller et al., 2011). Patients with spinal cord injury showed decreasing body ownership (Lenggenhager et al., 2012). Indeed, several studies of healthy subjects indicate incongruence between the proprioception associated with motor commands and visual feedback altered limb perceptions, such as pain heaviness extra limb and limb loss (McCabe et al., 2005; Foell et al., 2013). We previously showed that sensorimotor incongruence causes peculiarity (Katayama et al., 2016), and others showed that experimental modulation of sensory feedback reduces the accuracy of movements (Brandes et al., 2016; Lebar et al., 2017). These reports have clarified the influence of spatial sensorimotor incongruence on body perceptions, peculiarity and motor control. Therefore, it was not clear how much body perceptions, peculiarity and motor control were altered if temporal incongruence occurs between motor command and sensory feedback. In recent studies, experimental time delays between motor commands and sensory feedback disturbed body perceptions such as sensation of losing limb (Imaizumi et al., 2014; Osumi et al., 2018). The experimental paradigm manipulating temporal delay is considered possible to clarify, in detail, the degree of altered limb perception, peculiarity and movement disorders that are caused by temporal sensorimotor incongruence. However, no studies have investigated the neural mechanisms of these body perceptions, peculiarity and motor control on temporal sensorimotor incongruence. For this reason, the neural mechanisms of altered limb perceptions and movement disorders caused by temporal sensorimotor incongruence remain unknown.

Accordingly, we hypothesized that delayed sensory feedback from the motor command (temporal sensorimotor incongruence) precedes peculiarities that could cause altered limb perceptions and tested whether altered limb perceptions increase gradually with temporal incongruence. To clarify the related neural mechanisms, exact low-resolution brain electromagnetic tomography (eLORETA) was performed with independent component analysis (ICA), and maximally spatially independent spectral components were identified (Pascual-Marqui, 2007). The resulting voxel images for specified frequencies indicated regions and frequencies that tend to be active together (Aoki et al., 2015; Franz et al., 2016). The present experiments using eLORETA-ICA and an experimental feedback delay system that gradually increases temporal incongruence between motor commands and sensory feedback showed altered limb perceptions and clarified the related kinematic and perceptual changes and neural mechanisms.

## Materials and Methods

### Participants

Seventeen healthy students (age average 21.8 ± 0.8, 20–23 years old, 10 males and 7 females) all received and signed informed consent documentation before the study. Subjects were assessed using the Edinburgh handedness inventory test to confirm right handedness. All study protocols were conducted on the basis of the Declaration of Helsinki with the approval from the ethics committee of Kio University (approval number: H28-30).

### Experimental Feedback Delay System

**Figure 1:**
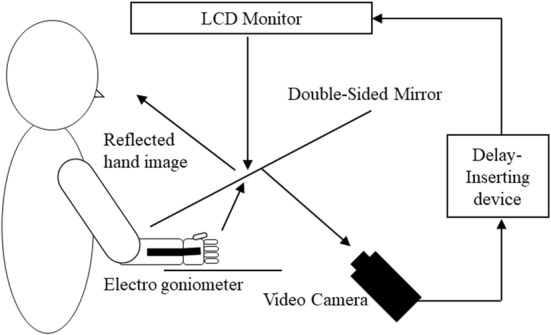
Demonstration of the experimental setup. Participants watched an image of their moving right hand that was delayed following their actual movement. The conditions of the visual feedback delay were 0, 150, 250, 350 and 600 ms delay conditions.

A feedback delay system was used to systematically distort sensory–motor integration as described previously (Shimada et al., 2010, 2014; Osumi et al., 2018; Figure 1). Using this system, visual feedback from movements can be delayed by set times, thus producing incongruence between movements and sensory feedback. After removing watches and jewelry, participants placed their right hand under a double-sided tilted mirror so that they could not see the hand. The hand was shown on the double-sided tilted mirror with a video camera (FDR-AXP35, Sony, Tokyo, Japan), and a video delay device (EDS-3306, FOR-A YEM ELETEX, Tokyo, Japan) was placed on the double-sided mirror to project the hand on an LCD monitor (LMD-A240, Sony). Because the hand was projected on the monitor, participants were not able to see their actual hands and only saw their hand in the mirror. The angles of the mirror were carefully measured before the experiment so the participants could see the image of the reflected hand as it was horizontally placed on the table. To achieve sensory feedback delays, we used a hardware device connected to the video camera and the monitor (EDS3305, ELETEX, Osaka, Japan) and performed experiments with delays of 0, 150, 250, 350 and 600 ms. Under these experimental settings, a unique sensory feedback delay of about 33.71 ms was determined using a time lag check device (EDD-5200, FOR-A YEM ELETEX, Tokyo, Japan).

### Protocol

Participants were instructed to perform periodic wrist movements at a frequency of 1 Hz. The pace of the movements was guided by the sound of a metronome in the practice sessions before the trial. Each participant performed their own pace and with their maximum ranges of extension and flexion and vice versa for 20 s under each of the delay conditions. We did not control the pace of periodic movements and observed natural variations in intervals and speeds of continuous movements. Participants viewed various delayed images and performed the sequence twice with 3 min breaks in between. All participants performed a total of 10 sequences (two replicates and five conditions). Delay conditions were pseudo-randomly applied to facilitate balance between participants.

### Behavioral Data Measurements

#### Subjective and Kinematic Data

Following the second sequence of all delay conditions, participants were asked to use the 7-point Likert scale (1, strongly disagree to 7, strongly agree) to rate their embodied changes according to the following statements: “I felt something peculiar during the movements,” “I felt something heavy during the movements,” and “I felt I was losing my hand during the movements.” We also measured wrist movements by attaching an electronic goniometer with a frequency of 1,000 Hz (Biometrics Model SG110A, Gwent, UK) to the back sides of subject’s right wrists and recorded data using the LabVIEW system. In addition to the relative cycle duration, the mean absolute deviation (MAD) of the relative cycle duration was used as an estimate of movement accuracy.

#### Electroencephalograph (EEG) Recording and Preprocessing

Electroencephalograph (EEG) signals were continuously recorded from 32 pre-amplified Ag/AgCl electrodes (Active Two, BioSemi, Amsterdam, Netherlands) embedded on an elastic cap (BioSemi) in accordance with the extended 10/20 system. In the BioSemi system, the ground electrode is replaced with a Common Mode Sense active electrode and a Driven Right Leg passive electrode. These two electrodes are located near Pz and POz electrodes and form a feedback loop that drives the average potential of the subject (the Common Mode voltage) as close as possible to the analog–digital converter reference voltage in the AD-box. The resulting EEG signals were digitized at a sampling rate of 1,024 Hz and were saved for online analyses. Saved EEG data were adapted online to a common average reference montage using multimodal EEG analysis software (EMSE Suite 5.4, Source Signal Imaging, La Mesa, CA, USA) with a scale band-pass filter from 1.0 Hz to 70.0 Hz. We also removed components that corresponded with ICA artifacts using EMSE Suite 5.4. These components included blinks, eye movements, facial movement activities and other body movements that cause artifacts.

#### EEG Networks Analysis

Prior to analyses, we excluded five participants who recognized excessive artifact mixing and conducted EEG network analyses on the other 12 participants. We analyzed EEG data online using eLORETA (free academic software available at http://www.uzh.ch/keyinst/loreta.htm, Pascual-Marqui et al., 2011). Specifically, eLORETA was used to compute cortical electrical distributions from scalp electrical potentials measured at each electrode site (Pascual-Marqui et al., 2011). The eLORETA method is a weighted minimum norm inverse solution with unique weights that allow precise localization for any point source in the brain. According to principles of linearity and superposition, we correctly localized arbitrary distributions, albeit with low spatial resolution. In the present eLORETA version, the solution space comprised 6,239 cortical gray matter voxels at a 5 mm spatial resolution in a realistic head model (Fuchs et al., 2002). Calculations were performed with the Montreal Neurologic Institute (MNI) average magnetic resonance imaging brain (MNI152) template (Fonov et al., 2011), with anatomic labels corresponding to Brodmann areas (BAs), which were used to compute the lead field. In imaging analysis, five epochs were extracted from the data for each condition (epoch duration, 20 s) and frequency analysis was performed. Data from frequency bands of interest (delta, 1.5–6.0 Hz; theta, 6.5–8.0 Hz; alpha 1, 8.5–10.0 Hz; alpha 2, 10.5–12.0 Hz; beta 1, 12.5–18.0 Hz; beta 2, 18.5–21.0 Hz; beta 3, 21.5–30.0 Hz; and gamma, 30.5–70.0 Hz) were calculated for intracerebral spatial analyses using the eLORETA method (Pascual-Marqui, 2002), which allows three-dimensional image displays of intracerebral neural activity. Values for each voxel in brain areas displaying neural activity under each condition were calculated as neural activity (current source density, μA/mm^2^ * 10^−3^) and were identified in terms of BA and MNI coordinates (Collins et al., 1995). Neural activity was then calculated as the global field power value (Pascual-Marqui, 2002) under each condition.

The linear inverse solution method eLORETA can reconstruct cortical electrical activities with correct localization from scalp EEG data (Pascual-Marqui et al., 2011; Aoki et al., 2013). We then performed eLORETA-ICA on the eLORETA localization images using the method described by Aoki et al. (2015) technical details can be found in Pascual-Marqui et al. (2011). Briefly, a mean localization image was initially calculated for each frequency band for each participant using data from each condition. These data were then concatenated, and eLORETA-ICA was performed to identify maximally spatially independent spectral components. The resulting components indicated regions and frequencies that tend to be active together in voxel images for each specified frequency (Aoki et al., 2015; Franz et al., 2016). The implementation of ICA in the eLORETA software with EEG data allows decomposition of cortical electrical activity, which is non-Gaussian, into independent components (ICs) in different frequency bands (Pascual-Marqui and Biscay-Lirio, 2011). Other decomposing methods, such as principal component analysis or correlation analysis, fail to do so with EEG data (Bell and Sejnowski, 1997; Hyvärinen and Oja, 2000; Mantini et al., 2011). Furthermore, eLORETA-ICA uses all frequency information from EEG data (Aoki et al., 2015; Franz et al., 2016). Thus, we used eLORETA-ICA to investigate neural mechanisms of altered limb perceptions due to sensorimotor incongruence.

### Statistical Analysis

#### Subjective and Kinematic Data

We confirmed normality of our data using the Shapiro-Wilk test. Subjective and kinematic data (MAD) were compared using the Friedman test, and when differences were significant at the <5% level, we further confirmed differences among the five conditions using Wilcoxon tests with Bonferroni correction. The standard level of Wilcoxon approval was set at 0.5%, and all calculations and statistical analyses were performed using SPSS version 25.0 (IBM Corp., Armonk, NY, USA).

### eLORETA-ICA in eLORETA

Intracerebral neural activities under each condition were compared using the statistics in eLORETA statistical nonparametric maps (Pascual-Marqui, 2007). In these analyses, neural areas showing significant differences in activity (*p* < 0.05) were colored, calculated and drawn. A significance threshold was determined using permutation tests with 5,000 permutations, log-transformed LORETA values (μV/mm^2^), and subject-wise normalization to correct for differences in baseline activity among all participants. These source localizations were then concatenated, and eLORETA-ICA was used to determine maximally spatially ICs. Components were then Z-transformed, and activation values of ≥3.0 were considered significant (Aoki et al., 2015).

### Correlation Analysis of eLORETA-ICA With Subjective and Kinematic Data

Spearman’s rank correlation coefficients were used to determine correlations between subjective and kinematic data in eLORETA-ICA, and significant differences in theta, alpha and gamma bands were calculated relative to data from the 0 ms time delay condition.

## Results

### The Effects of Visual Feedback Delays on Subjective Data

**Figure 2:**
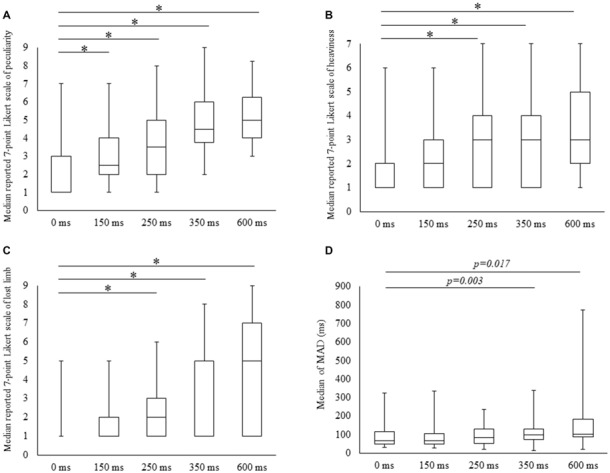
Degree of subjective and kinematic data detected under the five conditions. Boxes (median [interquartile range]) represent 7-point Likert scale scores for peculiarity **(A)**, heaviness **(B)**, lost limb **(C)** and median of mean absolute deviation (MAD; **D**). *Significant change (Wilcoxon test with Bonferroni correction to examine the differences among the five conditions *P* < 0.012, *p*-value adjusted for multiple comparisons).

Scores for all subjective items increased with time delays, with 7-point Likert scale values for peculiarities (mean ± SE) of 1.9 ± 0.3, 2.9 ± 0.3, 3.6 ± 0.4, 4.6 ± 0.4 and 5.0 ± 0.4 for 0, 100, 150, 250, 350 and 600 ms delay conditions, respectively. The results of Friedman tests confirmed the main effect (*χ*^2^ = 49.5, *P* < 0.01) and *post hoc* tests identified significant increases in peculiarity scores under all delay conditions compared with the 0 ms condition (*P* < 0.05; Figure 2A). Perceptions of heaviness in the 7-point Likert scale (mean ± SE) were 1.8 ± 0.3, 2.2 ± 0.3, 2.9 ± 0.4, 3.0 ± 0.5 and 3.5 ± 0.5 under delay conditions of 0, 100, 150, 250, 350 and 600 ms, respectively. Friedman tests confirmed the main effect (*χ*^2^ = 31.5, *P* < 0.01), and *post hoc* tests showed significant increases (*P* < 0.05) under 250, 350 and 600 ms delay conditions compared with the 0 ms condition (Figure 2B). The 7-point Likert scale (mean ± SE) values for the perception of limb loss were 1.4 ± 0.2, 1.9 ± 0.3, 2.6 ± 0.5, 3.5 ± 0.5 and 4.1 ± 0.6 under 0, 100, 150, 250, 350 and 600 ms delay conditions, respectively. Again, Friedman tests confirmed the main effect (*χ*^2^ = 31.4, *P* < 0.01), and *post hoc* tests showed significant increases (*P* < 0.05) for 250, 350 and 600 ms delay conditions compared with the 0 ms condition (Figure 2C).

### Effects of Visual Feedback Delays on Kinematic Data

MADs increased with durations of delays in the kinematic data, and these were (mean ± SE) 91.1 ± 16.2, 91.7 ± 17.2, 100 ± 15.5, 123.0 ± 20.1 for 350 and 162.6 ± 40.5 with delays of 0, 150, 250, 350 and 600 ms. The main effect was confirmed in Friedman tests (*χ*^2^ = 13.4, *P* < 0.01) and the post-test increase (*P* < 0.05) for the 350 ms delay condition was significant in comparison with the 0 ms condition. A similar tendency for the 600 ms delay condition was identified (*P* < 0.05; Figure 2D).

### Correlation Analysis of Subjective and Kinematic Data

**Figure 3:**
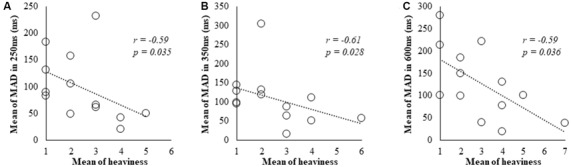
Correlation between subjective data and kinematic data for the 250, 350 and 600 ms delay condition. **(A)** Scatter plot showing a significant negative correlation between the heaviness and the MAD in 250 ms delay condition. **(B)** Scatter plot showing a significant negative correlation between the heaviness and the MAD in 350 ms delay condition. **(C)** Scatter plot showing a significant negative correlation between the heaviness and the MAD in 600 ms delay condition.

In Spearman’s correlation analysis of subjective and kinematic data, a significant negative correlation was observed between heaviness and MAD of cycle durations (*r* = −0.59, *P* = 0.035; *r* = −0.61, *P* = 0.028; *r* = −0.59, *P* = 0.036) under 250, 350 and 600 ms delay conditions, respectively (Figures 3A–C).

### eLORETA-ICA in eLORETA

**Figure 4:**
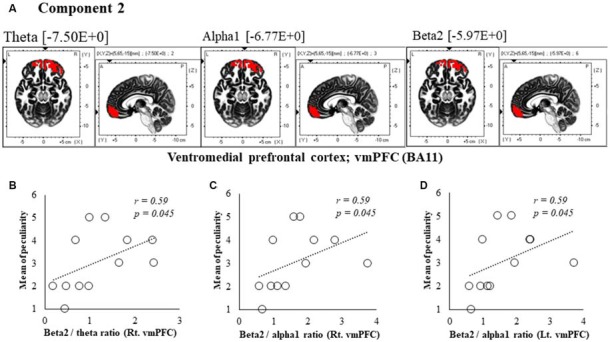
Correlation between 150 ms delay condition components and subjective data. **(A)** Component 2 corresponds to the ventromedial prefrontal cortex (vmPFC) in Theta, Alpha1 and Beta2 frequency band. The red colors indicate increased activity under the 150 ms delay condition. **(B)** Scatter plot showing a significant positive correlation between the peculiarity and the Beta2/theta ratio in Rt. vmPFC (*x* = 5, *y* = 65, *z* = −15). **(C)** Scatter plot showing a significant positive correlation between the peculiarity and the Beta2/alpha1 ratio in Rt. vmPFC (*x* = 5, *y* = 65, *z* = −15). **(D)** Scatter plot showing a significant positive correlation between the peculiarity and the Beta2/alpha1 ratio in Lt. vmPFC (*x* = −5, *y* = 60, *z* = −20).

**Figure 5:**
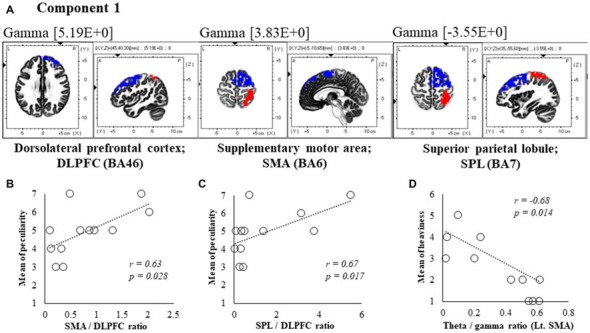
Correlation between 600 ms delay condition components and subjective data. **(A)** Component 1 corresponds to the dorsolateral PFC (DLPFC), supplementary motor area (SMA) and superior parietal lobule (SPL) in Gamma frequency band. The red colors indicate increased activity under the 600 ms delay condition. The blue colors indicate decreased activity under the 600 ms delay condition. **(B)** Scatter plot showing a significant positive correlation between the peculiarity and the Lt. SMA/Rt. DLPFC ratio in gamma band. **(C)** Scatter plot showing a significant positive correlation between the peculiarity and the Rt. SPL/Rt. DLPFC ratio in gamma band. **(D)** Scatter plot showing a significant negative correlation between the heaviness and the Theta/gamma ratio in Lt. SMA (*x* = −5, *y* = −10, *z* = 65). Rt. DLPFC (*x* = 45, *y* = 40, *z* = 30), Rt. SPL (*x* = 35, *y* = −55, *z* = 60).

**Figure 6:**
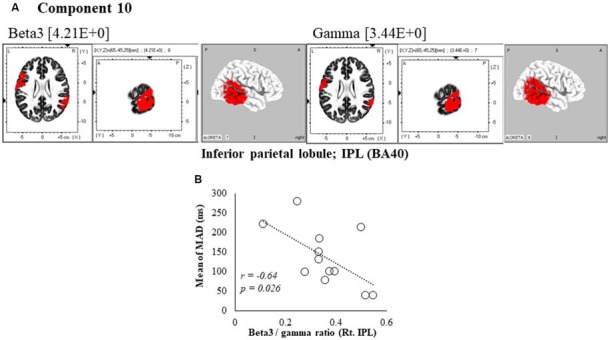
Correlation between 600 ms delay condition component and kinematic data. **(A)** Component 10 corresponds to the inferior parietal lobule (IPL) in Beta3 and Gamma frequency band. The red colors indicate increased activity under the 600 ms delay condition. **(B)** Scatter plot showing a significant negative correlation between the mean of MAD and the Beta3/gamma ratio in Rt. IPL (*x* = 65, *y* = −45, *z* = 25).

**Figure 7:**
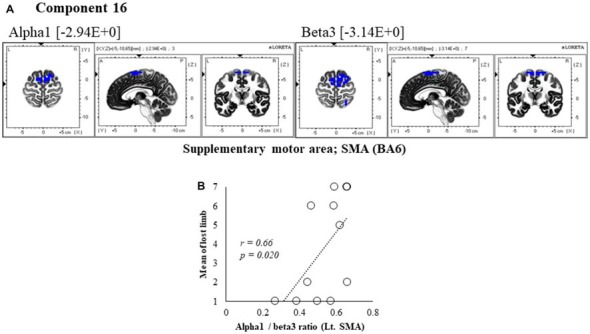
Correlation between 600 ms delay condition component and subjective data. **(A)** Component 16 corresponds to the SMA in Alpha1 and Beta3 frequency band. The blue colors indicate decreased activity under the 600 ms delay condition. **(B)** Scatter plot showing a significant positive correlation between the lost limb and the Alpha1/beta3 ratio in Lt. SMA (*x* = −5, *y* = −10, *z* = 65).

When comparing the 0 ms condition with the 150 ms delay condition, 13 components were extracted, and 8 of these differed significantly at 0 ms (*t* = 1.1, *P* < 0.05). In comparisons of the 150 ms delay with 250 and 350 ms delay conditions, no significant differences were found in the 16 extracted components (*t* = 0.2, *P* > 0.05; *t* = 0.2, *P* > 0.05). Comparisons of 0 and 600 ms delay conditions also led to the extraction of 16 components, and these were all significantly different between the conditions (*t* = 0.2, *P* < 0.01; Figures 4A, 5A, 6A, 7A).

### Correlations of eLORETA-ICA With Subjective and Kinematic Data

We extracted components that differed significantly between 150 ms and 600 ms time delays and the 0 ms condition in eLORETA-ICA. Subsequently, we correlated these with subjective and kinematic data as described in the following sections.

### Correlation Analysis of 150 ms Delay Condition Components With Subjective and Kinematic Data

In component 2, the neural activity of the bilateral ventromedial prefrontal cortex (vmPFC) BA 11 in were significantly higher under delayed vs. 0 ms conditions, as indicated by theta, alpha1 and beta2 bands (*P* < 0.05), and a cooperative relationship was observed between frequency bands. In particular, we identified a significant positive correlation between the right vmPFC’s beta2/theta ratio and peculiarity (*r* = 0.59, *P* = 0.045; Figure 4B). There was also a significant positive correlation between the bilateral vmPFC beta2/alpha1 ratio and peculiarity (*r* = 0.59, *P* = 0.045; Figures 4C,D). No significant correlations were found between the other components and subjective and kinematic data.

### Correlation of Components and Subjective and Kinematic Data Under 600 ms Delay Conditions

In component 1, neural activities in the gamma band left supplementary motor areas (SMA) BA6 and right dorsolateral PFC (DLPFC) BA46 were significantly lower under 600 ms delay conditions than under 0 ms conditions, and significantly higher neural activity (*P* < 0.05) was observed in the right superior parietal lobule (SPL) BA7, although a contradictory correlation was detected between the SMA, the DLPFC and the SPL. In further correlation analyses, a significant positive correlation was observed between peculiarities of the left SMA/right DLPFC ratio and the right SPL/right DLPFC ratio (*r* = 0.63, *P* = 0.028; *r* = 0.67, *P* = 0.017; Figures 5B,C). Finally, the theta/gamma ratio in the left SMA was significantly negatively correlated with heaviness (*r* = −0.68, *P* = 0.014; Figure 5D).

In the component 10, neural activity of the beta3 and gamma band right inferior parietal lobule (IPL) BA40 was significantly higher than in under the 0 ms condition, and a cooperative correlation was present between frequency bands. Moreover, a significant negative correlation was identified between MAD of delays, showing the accuracy of movements, and the right IPL’s beta3/gamma ratio (*r* = −0.64, *P* = 0.026; Figure 6B).

In component 16, neural activity of the alpha and beta3 band left SMA was significantly lower with 600 ms delays than with 0 ms delays, and the frequency bands we cooperatively correlated. The perception of limb loss was significantly positively correlated with the alpha/beta3 ratio in the left SMA (*r* = 0.66, *P* = 0.020; Figure 7B), whereas no significant correlations were found between the other components and subjective and kinematic data.

## Discussion

In this study, peculiarities were only provoked when the degree of time delay was 150 ms, and lost limb and heaviness were provoked as body perception changes after 250 ms. However, it was clarified that movement accuracy decreased after 350 ms. In other words, we suggested that body perception changes and movement disorders are different depending on the degree of temporal incongruence. Previous studies have demonstrated that patients with strokes or pathological pain suffer distorted limb ownership and an inability to perceive their affected limbs as a part of their bodies (Galer and Jensen, 1999; Frettlöh et al., 2006; Burin et al., 2015). Additionally, other studies showed a decrease of movements accuracy (Bank et al., 2015; Osumi et al., 2017). This our result may be useful for grasping the extent of temporal incongruence of the patient’s sensory movement by evaluating the change in body perception and movement disorders felt by the patient.

### The Effect of Visual Feedback Delays on Subjective Perceptions

All subjective scores were distorted with greater time delays between movement executions and sensory feedback. These peculiarities were provoked by 150 ms delays, before which disturbances of limb ownership and heaviness were reported by participants. Although these data suggest a causal relationship with sensorimotor incongruence (McCabe et al., 2005; Foell et al., 2013; Katayama et al., 2016), body ownership and heaviness are reportedly influenced by several cognitive factors (Flanagan and Beltzner, 2000; Synofzik et al., 2008). Accordingly, provoked peculiarity sensations and perceptions of distorted limb ownership and heaviness differed significantly between feedback delay times. These results suggest that peculiar sensations are distorted prior to the disturbance of limb ownership and heaviness.

### Effects of Visual Feedback Delays on Kinematic Data

MAD increased with visual feedback delays of >350 ms. Furthermore, there was significant negative correlation between heaviness and MAD under 250, 350 and 600 ms delay conditions. Under 150 and 250 ms delay conditions, no increases in MAD were observed regardless of peculiarity and lost limb perceptions, suggesting that heaviness modifications are related to increases in MAD.

It is established that movement accuracy is controlled by sensory feedback (Sperry, 1950; Wolpert et al., 1995) and experimental disturbances of sensory feedback reportedly reduce the accuracy of movements (Brandes et al., 2016; Lebar et al., 2017; Osumi et al., 2018). Additionally, subjective heaviness was altered with sensorimotor incongruence (Kuppuswamy et al., 2016). We thought that there was a similar mechanism between reduced movement accuracy and altered heaviness; hence, a significant correlation was found between heaviness and MAD. Therefore, we suggested that delays in visual feedback cause sensorimotor integration failure, increase heaviness and decrease movement accuracy.

### eLORETA-ICA Analysis in eLORETA

In eLORETA-ICA, components that changed significantly with delay conditions were only extracted in comparisons of the 0 ms condition with 150 and 600 ms delay conditions. These components comprised multiple frequency bands in various brain regions. Moreover, characteristic oscillations in the cortex network coexisted as multiple frequency bands and interactions between these were reported previously (Jensen and Colgin, 2007). Cross-frequency coupling has been observed in several brain regions, including the hippocampus, the PFC and the sensory cortex (Chrobak and Buzsáki, 1998; Buzsáki and Draguhn, 2004; Jones and Wilson, 2005; Lakatos et al., 2005; Mormann et al., 2005; Canolty et al., 2006). Some reports also show visual feedback delays in active and passive self-body movements using feedback delay systems similar to the present one (Shimada et al., 2010). In a previous study, active self-body movements were detected at rates close to 100% under 150 ms delay conditions, 10% under 350 ms delay conditions, and 50% and 95% under delay conditions of 250 and 350 ms, respectively. However, detection rates varied between subjects under 150 and 350 ms delay conditions (Shimada et al., 2010), suggesting that the present significant differences between the 0 ms and 250 and 350 ms delay conditions, in which components were not extracted, reflect differing ICs and varying detection rates between subjects. Yet because we did not assess detection rates, it is not clear that these differ between subjects under 250 and 350 ms delay conditions. This will be a point of clarification in future studies.

### Components That Relate eLORETA-ICA With Subjective and Kinematic Data

#### Components Related to Peculiarity

A positive correlation was found between the 150 ms delay condition component 2, where theta, alpha and beta2 bands increased in the vmPFC, which is involved in emotional processing (Northoff and Bermpohl, 2004). Similarly, peculiarities of beta2/theta and beta2/alpha1 ratios were observed. The beta band is considered a frequency band from the cerebral cortex (Spitzer and Haegens, 2017), whereas theta and alpha bands are closely related to subcortical structures, such as the thalamus (Sarnthein et al., 2003, 2005; Uhlhaas et al., 2008). Considering these studies, cortical activity of the vmPFC under 150 ms delay conditions was identified as a peculiarity of neural processing that occurred before the perception of limb loss. Unlike 150 ms delay conditions, neural processing was concurrently related to peculiarities in the 600 ms delay condition, with a positive correlation between the peculiarity and component 1 gamma band SMA/DLPFC and SPL/DLPFC ratios. The DLPFC specifically monitors conflict between motor commands and the ensuing sensory and perceptual consequences (Fink et al., 1999). Therefore, the SMA/DLPFC likely reflects the motor system, and the SPL/DLPFC reflects the sensory system. Furthermore, the gamma frequency band was reportedly related to sensorimotor integration during visuomotor tasks (Roelfsema et al., 1997). Hence, peculiarities in the 600 ms delay condition may be related to the cortical process from integration of sensorimotor information to monitoring of congruency. These results suggest that the present occurrences of peculiarities follow brain activities that are related to emotional processing and sensorimotor integration.

#### Lost-Limb-Related Component

In component 16 of the 600 ms delay condition, the alpha/beta3 ratio in the SMA was positively correlated with the perception of limb loss. The alpha oscillation in SMA was previously associated with movement preparation or with images (Pfurtscheller and Aranibar, 1979). Furthermore, beta bands have been frequently associated with sensorimotor network processing (Hari and Salmelin, 1997; Pfurtscheller and Lopes da Silva, 1999; Brovelli et al., 2004). Considering that incongruence between motor commands and sensory feedback provoke the sensation of limb loss, the present alpha/beta3 ratios indicate that EEG activity is related to limb loss. Therefore, we suggested that the alpha/beta3 ratio in the SMA reflects neural processing related to limb loss.

#### Heaviness-Related Component

In comparisons between sequences of the motor outflows motor intention and command to muscles, peripheral signals from muscle spindles are thought to induce the sensation of heaviness (Luu et al., 2011). In component 1 of our 600 ms delay conditions, the theta/gamma ratio was significantly correlated with heaviness in the SMA. Numerous reports show cross-frequency coupling of gamma and theta bands (Jensen and Colgin, 2007; Friese et al., 2013). Furthermore, the SMA was reportedly associated with high motor control (Picard and Strick, 1996), and in another study, the gamma band increased in the SMA during voluntary exercise (Ball et al., 2008; Cheyne et al., 2008). Therefore, we suggest that higher motor cortical activity, such as that reflected by SMA gamma bands, is related to the sense of heaviness.

#### Movement-Accuracy-Related Component

In component 10 of the 600 ms delay condition, beta3/gamma ratios in the right IPL were significantly correlated with MAD. Accordingly, the right IPL was shown to integrate sensory feedback information and efference copy information (Murata and Ishida, 2007) and was increasingly activated when these information sources were incongruent (Fink et al., 1999; Farrer and Frith, 2002; Farrer et al., 2003; Balslev et al., 2005; Shimada et al., 2005; Katayama et al., 2016). The beta band is traditionally associated with motor processing (Hari and Salmelin, 1997; Pfurtscheller and Lopes da Silva, 1999; Brovelli et al., 2004), whereas the gamma band is considered a frequency band with roles in temporal binding and perceptual integration (Engel and Singer, 2001). In accordance with these studies, we relate the gamma band with both motor and sensory processing. Because sensory feedback is considered important in motor control (Sperry, 1950; Wolpert et al., 1995), beta3/gamma ratios that were related to sensorimotor integration were correlated with movement accuracy. Therefore, we suggest that beta3/gamma ratios in the IPL reflect neural processing related to movement accuracy.

Taken together, in this study, we analyzed the measured EEG data using eLORETA-ICA and revealed that the active frequency band of the area-related limb perception is change due to temporal sensorimotor incongruence. In other words, gamma band’s activity in SMA is related to peculiarity with DLPFC. Furthermore, alpha/beta3 ratio of SMA is related to limb loss and theta/gamma ratio is related to heaviness. In the parietal association area, gamma band’s activity in SPL is related to peculiarity with DLPFC. Also, we were revealed that IPL’s beta3/gamma ratio is related to movement accuracy. In a previous study, normal subjects were asked to either imagine or execute auditory-cued hand movements. Therefore, the study showed that supplementary motor and posterior parietal areas were more engaged in mental simulation (Gerardin et al., 2000). Furthermore, parietal area’s neurons appeared to combine visual and somatosensory signals in order to monitor the configuration of the visual and somatosensory information from limbs (Graziano et al., 2000). Therefore, these brain regions are reportedly involved in all processes from multisensory integration to motor programing. These results suggested that rather than a single activity in a local area, other area’s network such as abnormalities of multifrequency band activity was related to neural mechanism of altered limb perception and decrease movement accuracy caused by temporal sensorimotor incongruence. Neuroimaging studies have demonstrated that the function of these brain areas decreased on patients with strokes or pathological pain (Gieteling et al., 2008; Feinberg et al., 2010). Our results suggest that abnormalities in these areas contribute to neural mechanisms that modify altered limb perceptions and movement accuracy.

### Limitations

In the present study, we only investigated four arbitrary time frames, including the 150-ms delay condition delay. Our data warrant further experiments with more detailed feedback delay conditions to accurately clarify time frames in which changes in physicality and movement control occur. Secondly, we could not control the pace of movements to observe modifications of spontaneous movements. We also failed to unify numbers of wrist joint movements performed over 20 s without controls, and these potentially influence perceptions of heaviness. Thus, our results require verification by unifying the numbers of movements. Thirdly, significant differences in extracted components between 250, 350 and 0 ms delay conditions remain unexplained and could be resolved with measurements of differences in detection rates between subjects. Fourth, the present EEG analyses do not indicate the direction of information flow between brain regions. Thus, to clarify whether information flows between brain regions are related to clear altered limb perceptions and movement accuracy, effective connectivity analyses are necessary. Finally, our subjects were healthy and may not emulate the emotional mechanisms that prevail in brains of stroke and pathological pain patients who have symptoms of actual peculiarity or altered limb perceptions.

## Conclusion

In this study, we investigated the hypothesis that sensory feedback on motor commands causes temporal inconsistencies by triggering peculiarities prior to altered limb perceptions, and with longer discrepancies, altered limb perceptions become clearer. Potentially, peculiarity is caused prior to lost limb and heaviness, as indicated by our clarification of the neural processing related to peculiarities, altered limb perceptions and movement accuracy.

## Author Contributions

All authors conceived the study, performed the experiment and approved the final version of the manuscript. OK, TT, MO and TK analyzed the results. OK, MO and SM wrote the manuscript.

## Conflict of Interest Statement

The authors declare that the research was conducted in the absence of any commercial or financial relationships that could be construed as a potential conflict of interest.
